# WebEase: Development of a Web-Based Epilepsy Self-Management Intervention

**Published:** 2008-12-15

**Authors:** Colleen DiIorio, Cam Escoffery, Katherine A. Yeager, Archana Koganti, Elizabeth Reisinger, Archana Koganti, Frances McCarty, Thomas R. Henry, Elise Robinson, Rosemarie Kobau, Patricia Price

**Affiliations:** Rollins School of Public Health, Emory University; Emory University; Emory University; Emory University; Emory University; Grady Memorial Hospital; Georgia State University, Atlanta, Georgia; University of Minnesota, Minneapolis, Minnesota; Harvard University, Boston, Massachusetts; Centers for Disease Control and Prevention, Atlanta, Georgia; Centers for Disease Control and Prevention, Atlanta, Georgia

## Abstract

People with epilepsy must adopt many self-management behaviors, especially regarding medication adherence, stress management, and sleep quality. In response to the need for theory-based self-management programs that people with epilepsy can easily access, the WebEase Web site was created and tested for feasibility, acceptability, and usability. This article discusses the theoretical background and developmental phases of WebEase and lessons learned throughout the development process. The WebEase research team developed content for the Web site on the basis of social cognitive theory, the transtheoretical model of behavior change, and motivational interviewing. Formative research and development of the WebEase program included a literature search, computer use survey, a focus group, and review by content experts and consumers. The program has 2 main components: 1) the modules, which provide a tailored opportunity for learning, reflection, and goal setting, and 2) MyLog, a place to enter daily information.

## Introduction

More than 2.7 million Americans have epilepsy (1), and living with epilepsy requires adopting behaviors to control seizures and manage the consequences of having a seizure disorder (2,3). Perhaps the most essential health behavior for people with epilepsy is taking antiepileptic drugs. Although up to 70% of people can control seizures with medication (4), 30% to 60% do not take their antiepileptic drugs as prescribed (5,6) and put themselves at risk for seizures (7). In addition to taking medications, people with epilepsy must identify and avoid or control factors that trigger seizures. Stress and sleep deprivation have been identified as the 2 most common precipitants for seizures (8-14).

Studies of people with chronic disorders, including epilepsy, show that theory-based programs can foster self-management practices, such as taking medications as prescribed and managing stress and sleep (15-24). In addition, these programs enhance knowledge about the condition, promote positive attitudes, and improve quality of life (15,16,18). Despite the success of programs like Sepulveda Epilepsy Education (21) and Modular Service Package Epilepsy (22,23), implementing them can be challenging. Most are delivered by trained facilitators in 1 or more sessions, and participants may be required to attend multiple sessions to benefit. Barriers include the need to train facilitators or health educators to conduct a program, the costs of marketing and presenting it, scheduling problems, and lack of transportation for participants, who typically have driving restrictions.

The US Department of Health and Human Services recognizes the potential of technology to help people with chronic disorders overcome such barriers to better manage their health (25). For example, the eHealth Behavior Management Model is a promising Internet-based behavior change model for asthma management, preventing human immunodeficiency virus infection, and parent-child nutrition education (26). The online version of the Chronic Disease Self-Management Program led to improvements in health outcomes among participants with chronic disease (heart disease, lung disease, or type 2 diabetes) (27). A recent review examining the effectiveness of Web-based vs non–Web-based interventions for behavior-change outcomes (eg, increased exercise time, weight loss maintenance) found improvements for people who used Web-based interventions (28).

In 2005, the Centers for Disease Control and Prevention's Epilepsy Program recognized the dearth of accessible, theory-based self-management programs for people with epilepsy and provided funding to develop an Internet-based epilepsy self-management program. WebEase (Web Epilepsy Awareness Support and Education) was created in response to this initiative. In this article, we discuss the development of WebEase, including formative research and the development of the Web site. We also discuss challenges to the development of a theory-based self-management program in a Web interface and lessons learned.

## Phase 1: Formative Research

### Literature review

The WebEase development team was composed of an interdisciplinary group of investigators and staff with expertise in epilepsy treatment and care, health education, behavioral research, Web design and development, and program evaluation. The first task the team undertook was to review self-management programs and Web sites. This review provided useful information on the content, delivery, and teaching strategies used in Web site development. Additionally, we specifically reviewed program Web sites that used interactive applications of the constructs from the 3 theoretical models — transtheoretical model of behavior change (29,30), social cognitive theory (31), and motivational interviewing (32) — used to develop WebEase. Most chronic disease Web sites, including those specifically for epilepsy, provided considerable content about the condition, primarily through fact sheets. Web sites varied in the type of additional content available, and some provided feedback and links to resources (33); however, interactive components, including those based on behavioral theories, were often limited.

### Computer use survey

To assess the potential market for an Internet-based epilepsy program, we gathered information about computer use from people with epilepsy and their caregivers. The survey was disseminated through 2 epilepsy Web sites, and a paper-and-pencil survey was distributed to patients attending 2 epilepsy clinics in the Atlanta metropolitan area. For both methods, participants were asked to sign a consent document. The survey results showed that more than 95% of the online group and more than 60% of the clinic group had access to computers and the Internet (34). More than 99% of the online group and more than 57% of the clinic group used the Internet to find health information. Most people (73%) reported that they were likely to use an Internet-based self-management program to control their epilepsy. Approximately 43% reported searching on the Internet for general information about epilepsy, 30% for information about medication, 20% for information about specific types of epilepsy, and 20% for information about treatment. From this survey, we learned that our potential study participants had access to computers and the Internet. The respondents also said they desired epilepsy-specific information and were receptive to an online source for information on how to manage their epilepsy (34).

### Focus group

We conducted a focus group with 6 people who had epilepsy to discuss in more detail some of the issues raised by the survey and to verify its findings. As revealed by the survey, most participants used computers to obtain health information and most expressed interest in an online self-management program. Focus group participants also provided information about the 3 content areas — medication, stress, and sleep management — that would later be incorporated into WebEase. Institutional review board approval was obtained, and informed consent was given for both the computer use survey and the focus group.

## Phase 2: Development of WebEase

WebEase components include 3 core modules, a daily log, discussion boards, fact sheets, quizzes, daily poll questions, and links to online resources.

### Modules

The modules for medication, stress, and sleep management are the core of the WebEase program. By working through the modules, participants assess their status in each of these 3 self-management areas, reflect on their behavior, and create a plan for change or to maintain their behavior if no change is required. Each module is composed of 5 submodules, which represent the 5 categories of the stages of change: precontemplation, contemplation, preparation, action, and maintenance. Each submodule is composed of 3 sections: an introductory assessment section (Looking at My Medications), a section devoted to thinking about current behavior (Thinking About My Medications), and a section on goal setting and planning (Planning the Next Steps).

The introductory section of a module is the same for everyone. It provides information from participants' daily log (MyLog) and includes a series of questions about their behavior — medication taking, stress, or sleep. Feedback is given to reflect and summarize answers. The information obtained from answers to the questions is used to assign the participant to 1 of the 5 transtheoretical model stages. The second section includes information and activities that vary depending on assigned stage. The focus of the precontemplation stage is supporting positive attitudes toward the behavior and encouraging the participant to think about it; contemplation focuses on comparing the benefits and costs of the behavior and beginning to think about strategies to support it; preparation stresses strategies to support the behavior and the resources this requires; the action phase assesses confidence in one's ability to continue the behavior and the required resources; and maintenance deals with life changes that may interfere with the behavior and contemplating how it fits in one's life. Activities may include 1 or more of the following theory-based strategies: benefits, costs, previous successes, barriers, confidence in making changes, values, reasons for not changing, how life would differ if behavior did or did not change, resources and skills needed, and identification of a support person. The content throughout the modules is delivered by using motivational interviewing principles.

Links to fact sheets and other information resources are included. This information prompts a participant to learn about the behavior, strategy, or skill at the time it is presented. Links are also presented to images and audio files that show participants who tell their stories about dealing with challenges related to the behavior.

In the third section, Planning the Next Steps, the participant has the choice of making a plan of action that includes specific performance goals. Those in the precontemplation, contemplation, and preparation stages first complete a "readiness scale" to assess how prepared they are to make changes, create strategies, and commit to goals. After receiving feedback on their answers, participants are asked if they are ready to take the next step and make a plan. If so, the module takes them through the process of creating a goal and identifying potential barriers and useful strategies to overcome them. Those in the action and maintenance phases also have the opportunity to create goals and a plan to change or maintain their current behavior.

### MyLog

MyLog was designed so that participants can enter daily information about their medications, seizures, stress, and sleep. These data are entered into a database, and feedback is given in 3 ways. First, a text summary of information entered gives averages for each behavior. Second, a set of graphs presents the information visually over a 7-day period. Both the summary and the graphs can be printed. Third, some information entered into MyLog is used within the modules.

### MyVoice

MyVoice is a series of discussion boards that provides a forum for sharing information and fostering relationships. Discussion boards for the 3 main topics are available to participants during the entire program. They may post questions, respond to one another, and share experiences related to medication, stress, and sleep. During the pilot study, a nurse logged onto the site each day to monitor discussions and identify problems that would require referral to a health care professional.

### Fact sheets, quizzes, daily poll questions, and Web resources

Several components of the program were designed to increase knowledge about medication, stress, and sleep management and were supplemental to the core modules. Fact sheets were developed on each of the 3 areas and were available for participants to read at their leisure throughout the program. In addition, short quizzes and daily poll questions on each topic area were designed to engage participants to learn about epilepsy. Web resources for epilepsy, including the Epilepsy Foundation, the Centers for Disease Control and Prevention, and Epilepsy.com, were linked to encourage participants to learn more about self-management.

## Phase 3: Review and Revision

The design process of the WebEase program used an iterative evaluation process recommended for interactive interventions (35) and commonly used in the development of Internet-based programs (36-38). The process consisted of a review by content experts and members of the target audience to assess feasibility, accuracy, and completeness of the content and to obtain initial reactions to the program. A pilot study of the final product was conducted to assess the usability and acceptability of the program (39).

### Review by content experts

Four nurses with expertise in the care of people with epilepsy, epilepsy self-management research, and theory-based behavioral interventions served on the content expert panel. At each stage of development, these experts reviewed materials and provided comments and suggestions on aspects such as accuracy of content, organization of information, ease of understanding and use, application of theory, relevance, and acceptability. For each review, the experts completed an evaluation form with questions specific to the content, acceptability, usability, and appropriateness of the information. In response to the experts' comments and suggestions, many changes were made to clarify and enhance the content and improve its presentation and usability.

### Review by consumers

Two people with epilepsy assisted the team by evaluating the acceptability and usability of the Web site during the development phase. After each module was created, these consumers came to our office individually and worked through the site. They logged onto WebEase, and as they navigated it, they told us what they were doing, why they were doing it, what they liked, and what problems they had. Two team members observed each consumer and took notes on comments about content, layout, navigation, relevance of materials, educational soundness, appeal, understanding of key messages, appropriateness of graphics, ease of use, problems encountered, and recommendations for change. The participants also completed a short survey on the amount of material, the site's organization and layout, images, their general opinion of the site, and what they liked, disliked, and would change. The participants' feedback led to several revisions of text, images, and layout.

### Pilot study

Thirty-seven people with epilepsy agreed to participate in the study to evaluate the acceptability and usability of WebEase and provide an initial test of its behavioral objectives. Participants were recruited from 2 epilepsy clinics. After completing the pretest, participants were able to use each module for 2 weeks. At the end of the 6 weeks, they completed a posttest. The procedure and outcomes of the pilot study are described elsewhere (39).

## Lessons Learned

WebEase was developed and tested during a 2-year period. The extensive work required for its development and testing was facilitated by a team of content, process, and programming experts. The diversity of expertise and skills was critical to the success of the project. During these 2 years, 3 students assisted. They supported the team by aiding in content development, Web site review and revision, participant recruitment, orientation, and data analysis.

The primary challenge was communication between Web programmers and content developers. The 2 groups were in 2 different offices in Atlanta and often did not fully understand each other's activities. To facilitate communication, we had several working sessions in which the content developers presented theories to be applied in the program and described how they wanted the constructs and activities translated online, and the programmers offered options to accomplish these ends. Communication was also facilitated by a team member with extensive experience in Web design who spoke the "language" of both groups. Revising the site was often tedious and time consuming. To facilitate the revision process, a content developer and a programmer reviewed the components together while the programmer made changes. This strategy was successful but unfortunately was not implemented until late in the development phase.

Our experience confirms the adage that project development always takes longer and costs more than was planned and budgeted. The first 6 months of the program went as planned; however, we were not satisfied with the staging items or the incorporation of motivational interviewing principles in the first version of the site. The overall plan, algorithms, and content were extensively revised. These changes required text to be rewritten for all 3 modules and sections to be reprogrammed. The new versions of the modules were more complex, with more strategies for change and more choices, which resulted in more branching. The team required an additional 6 months to make the changes and refine the modules.

For the content development team, incorporating theory-based concepts was often challenging. The overall objective of the program was to use proven behavioral change strategies to promote epilepsy self-management behaviors. Thus, the program could not be limited to educational information. Rather, we needed to design ways to encourage reflection on the behavior and to motivate change. The transtheoretical model, social cognitive theory, and motivational interviewing strategies were designed primarily for face-to-face encounters. The Web interface presented the challenge of trying to use these strategies to make the site interactive in the absence of face-to-face contact. In addition, giving feedback to some comments entered into the program proved difficult without understanding the context in which they were made. Feedback that involved simple reflection, such as repeating the participant's words, was easy to program. More complex feedback, however, required algorithms that accommodated the various general statements that were possible.

Based on our experience developing WebEase and the lessons we learned throughout the process, we offer the following recommendations for similar projects. Overall, we recommend that a project's timeline include sufficient time for development and unanticipated revisions. Because feedback on content development and programming is an iterative process, a member of the content development team and the programmer should work side by side at designated times to facilitate communication and troubleshooting. Additionally, the Web site should be designed so that the content development team can make minor revisions, allowing the programmer to focus on broader program issues. Finally, we recommend that developers be willing to allow some flexibility in applying the theoretical frameworks behind their program. As mentioned above, some of the motivational interviewing components did not easily translate to an Internet format without a facilitator. Along with creative incorporation of theoretical constructs, future programs could also make use of other online tools, such as a synchronous, interactive chat. Overall, the initial results are encouraging, and continued development of WebEase can facilitate education and strategies to manage epilepsy for a population with barriers to accessing these services in person. In developing and evaluating this online self-management program, we hope to increase self-management skills and improve the quality of life of people with epilepsy.
